# Supplementary material for: Cyst-Wall-Protein-1 is fundamental for Golgi-like organelle neogenesis and cyst-wall biosynthesis in Giardia lamblia
Source: Nat Commun. 2016 Dec 15;7:13859. doi: 10.1038/ncomms13859 (PMC5171811; doi:10.1038/ncomms13859)
Supplement: Supplementary Information — Supplementary Figures and Supplementary Tables. [file ncomms13859-s1.pdf]

Supplementary information for Ebneter et al., « Cyst-Wall-Protein-1 is fundamental for Golgi-like organelle neogenesis and cyst-wall biosynthesis in *Giardia lamblia* ».

Supplementary figure 1

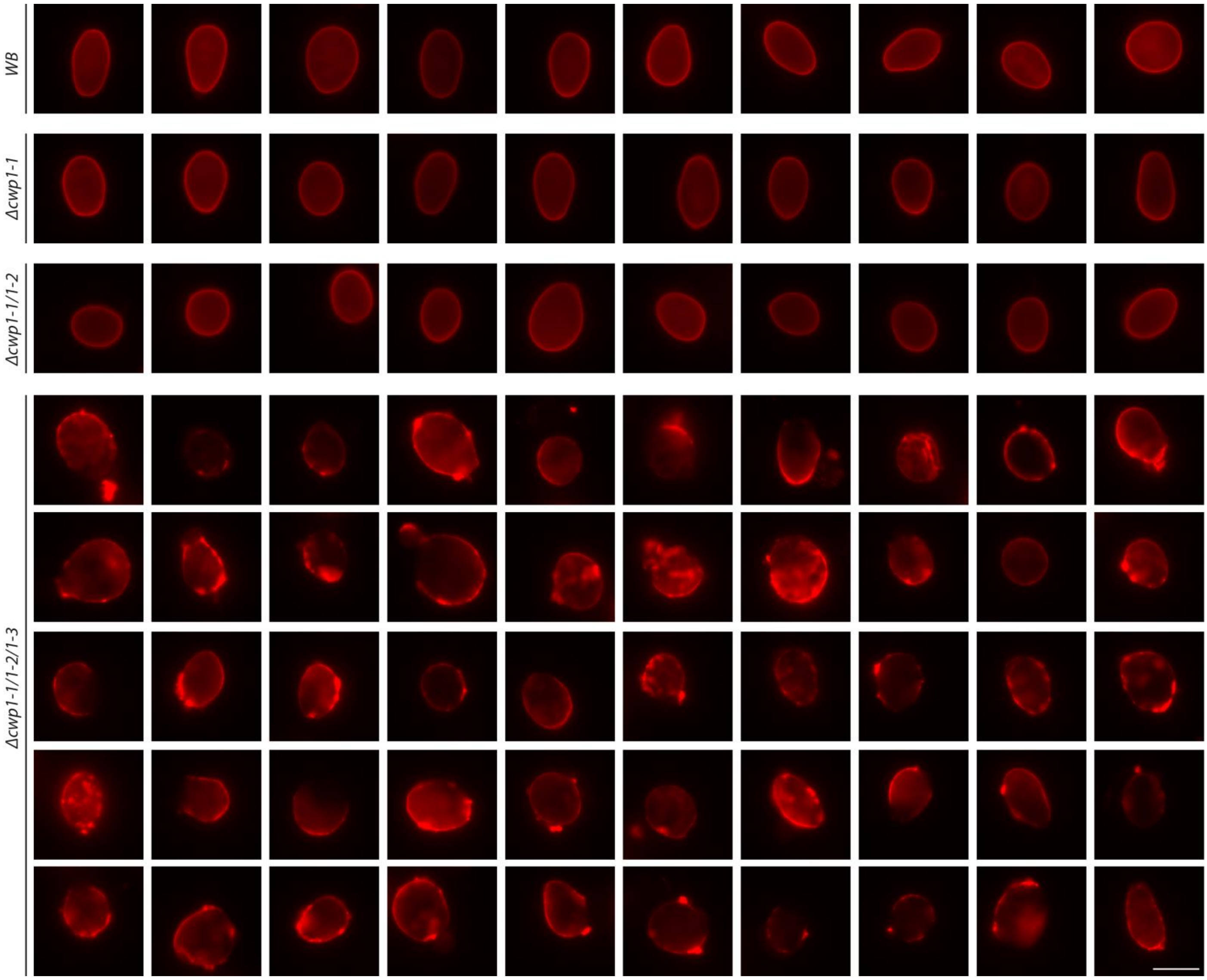

Supplementary Figure 1.

Cysts depleted of three out of four alleles present abnormal CWP1 deposition and distribution

Immunofluorescence analysis of wildtype (WB) cysts and cysts depleted of either 1 (*Δcwp1-1*), 2 (*Δcwp1-1/1-2*) or 3 (*Δcwp1-1/1-2/1-3*) *CWP1* alleles out of 4 highlights gross aberrations in the subcellular distribution of CWP1 only in *Δcwp1-1/1-2/1-3* cysts. Scale bar: 10 µm.

Supplementary Figure 2

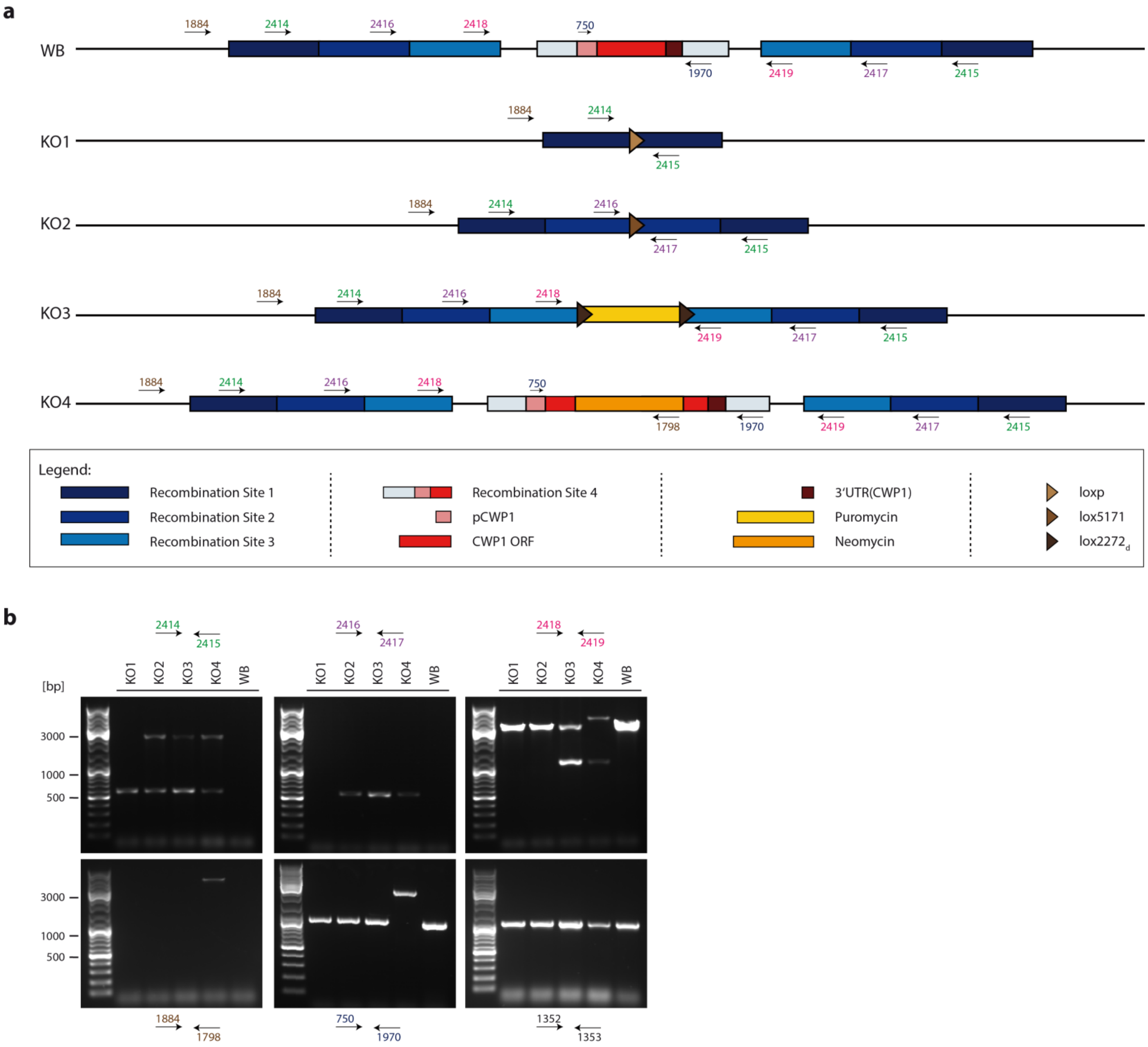

Supplementary Figure 2.

PCR analysis of genomic DNA to monitor sequential loss of intact CWP1 loci

(**a**) The upper scheme illustrates the status of the *CWP1* locus in WB-C6 cells and in progressively knocked-out lines (KO1 to KO4). (**b**) Genomic DNA from each line was extracted and analysed by PCR using oligonucleotide pairs with matching colors to produce unique DNA fragments. Amplification of a control locus (GGD gene model Gl50803_17161) was performed with oligonucleotides 1352 and 1353 (refer to Supplementary Table 1 for sequences). Marker lanes are labelled for main size bands (in bp).

Supplementary Figure 3

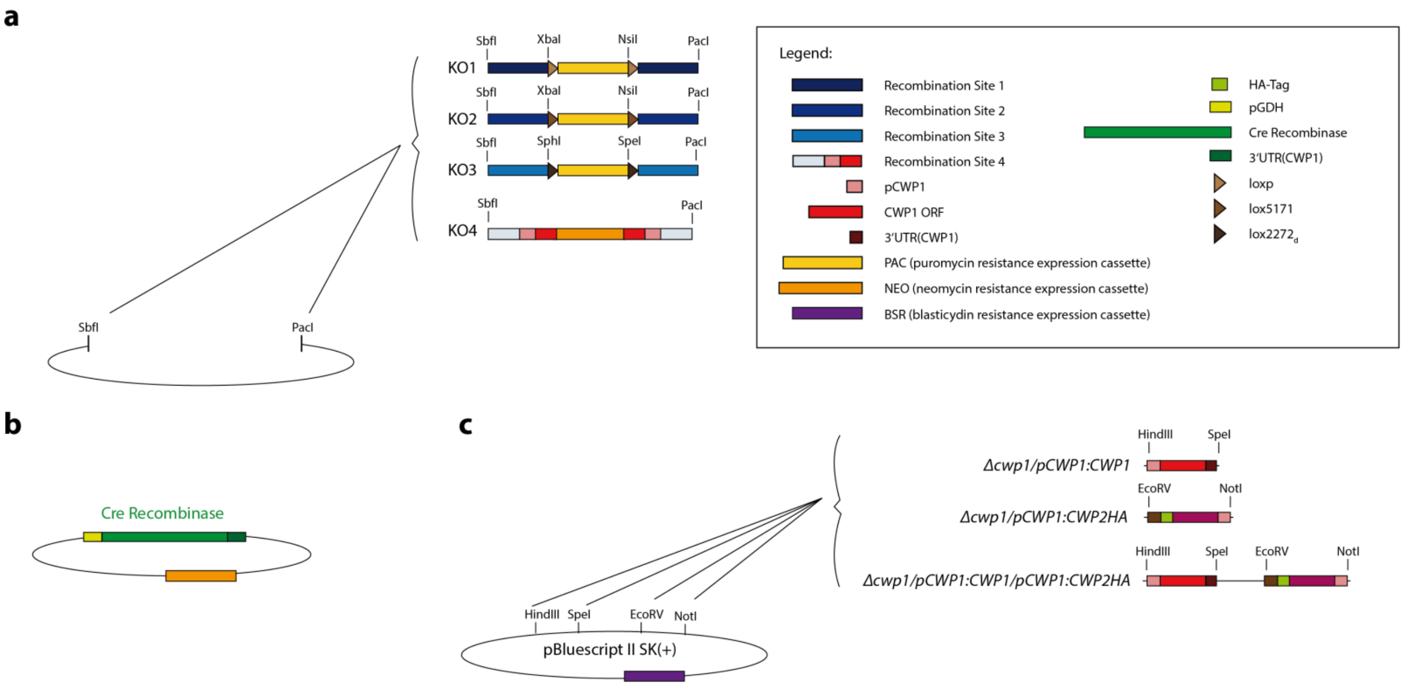

Supplementary Figure 3.

***Overview of plasmids constructed for the generation and complementation testing of line* Δcwp1**

(**a**) Plasmids CWP1KO1 to CWP1KO4, each harbouring a "floxed" puromycin resistance expression cassette (*PAC*) and 1000bp-long recombination sites homologous to DNA regions surrounding the genomic *CWP1* allele. Enzyme restriction sites used for engineering of the plasmids are indicated. (**b**) Plasmid encoding for constitutively-expressed Cre recombinase carrying a neomycine resistance expression cassette (*NEO*). (**c**) Plasmids coding for blasticidin resistance for expression of CWP1 and of an HA-tagged CWP2 reporter to monitor encystation in the absence of CWP1.

Supplementary Figure 4

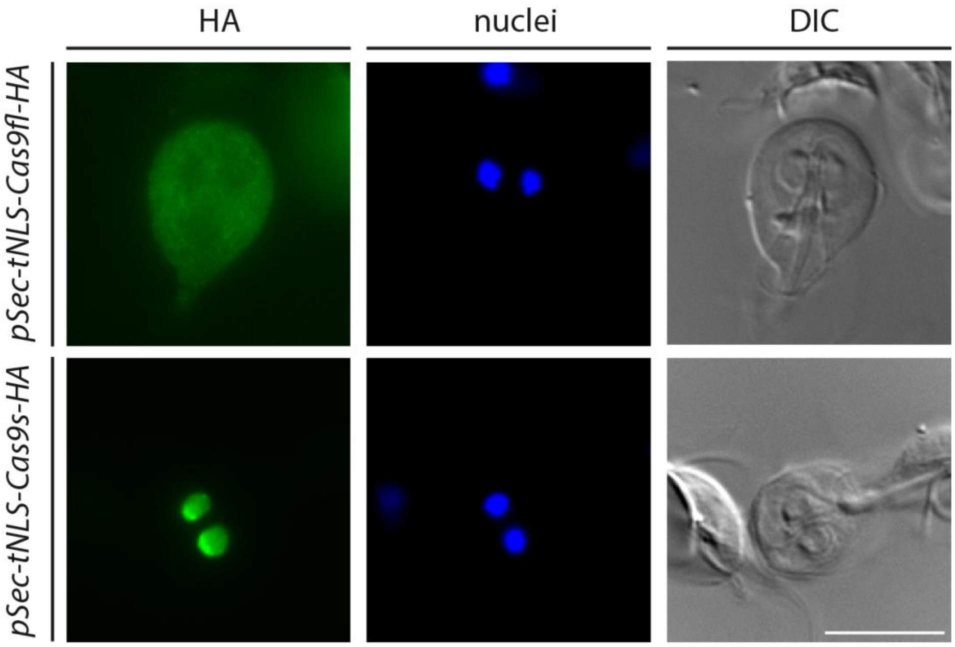

Supplementary Fig 4.

A truncated variant of S. pyogenes Cas9 can be targeted to the G. lamblia nucleus

Full length (*ca.*4250 bp**)** nuclear-targeted *S. pyogenes* Cas9 accumulates in the cytosol of transgenic *G. lamblia* cells (line pSec-tNLS-Cas9*fl*-HA). However, a similarly nuclear-targeted *ca.*1000bp truncated Cas9 variant is correctly deposited in the nuclei of expressing cells (line pSec-tNLS-Cas9*s*-HA). Scale bar: 10 µm.

Supplementary Figure 5

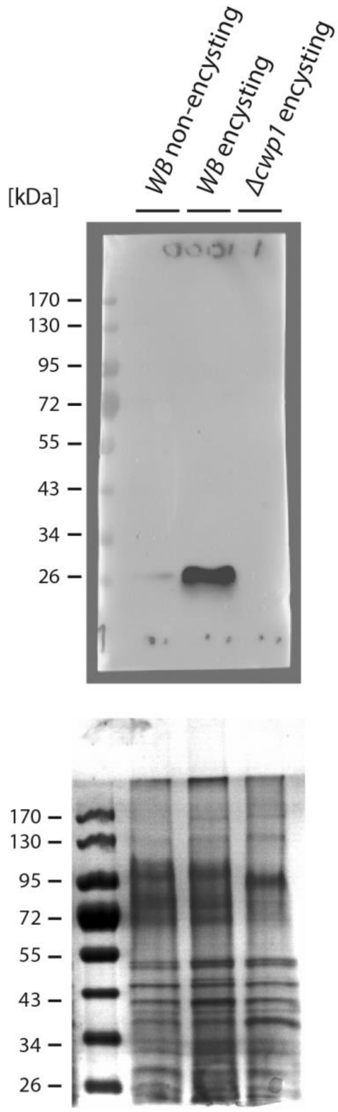

Supplementary Fig 5.

Immunoblot analysis of line Δcwp1

Uncropped, full view of the immunoblot and the Coomassie-stained protein gel shown in main figure 1d. A strong signal for CWP1 is visible in extracts derived from encysting WB cells, whereas no signal is obtained with *Δcwp1*-derived material. Marker lanes are labelled for main size bands (in kDa).

Supplementary Table 1

Oligonucleotides used for construct synthesis, genomic DNA, 3' RACE and RT-PCR analysis

**Oligonucleotide number**	**Oligonucleotide name**	**Sequence 5'-3'**	**Application**
750	CWP-s-NheI	ATGCTA GCCTAGCCACGCATGGGCTGT	amplification of allele *cwp1-4* from genomic DNA
1040	k-adaptor	CCGGAATTCGGTACCTCTAGA	3' RACE: amplification step
1045	k-anchorV	CCGGAATTCGGTACCTCTAGA(T18)V	3' RACE: reverse-transcription step
1080	CWP11SpeI	CGACTAGTATGATGCTCGCTCTCCTTG	RT-PCR: amplification step for *cwp1*
1352	Tom40_Spe_for	gc ACTAGT ATGCCCTTTCCTGGTCT	amplification of a control locus from genomic DNA and amplification step for GGD gene model Gl50803_17161 (*tom40*) in RT-PCR
1353	Tom40_SbfI_rev	gc CCTGCAGG GC CTTGTGATTGCTCGAAGA	amplification of a control locus (GGD gene model Gl50803_17161 ; *tom40*) from genomic DNA
1849	GDH5'UTR Xba for	GC TCT AGA GAA GCG CTG ACC ACA AAT AAC GC	cloning of the promoter and 5'UTR of giardial *GDH*
1850	GDH5'UTR Nsi rev	CG ATGCAT GGA TTT TAA AAT CTG GGG CGC C	cloning of the promoter and 5'UTR of giardial *GDH*
1865	CWP1KO1Sbf for 1	GC CCTGCAGG TTG ACG GAG ATA GGG GAC TGC TC	cloning of fragment RS1 for construct CWP1KO1
1866	CWP1KO1Xbarev 1	CG TCTAGA TTA TCC CTG CGG AAG AAA TTC AAC TAT GAT	cloning of fragment RS1 for construct CWP1KO1
1867	CWP1KO1Xbafor 2	GC TCTAGA ATAACTTCGTATAATGTATGCTATACGAAGTTAT GAAGCGCTGACCACAAATAAC	cloning of "floxed" *PAC* cassette for construct CWP1KO1 (loxP site is underlined)
1868	Puro3'UTR Nsi rev	CG ATGCAT G AAT TCG AGC TCG GTA CCA G	cloning of "floxed" *PAC* cassette for construct CWP KO1
1869	CWP1KO1Nsifor 4	GC ATGCAT ATAACTTCGTATAATGTATGCTATACGAAGTTAT ATGCACGATAGTACGCTCACTAGAGG	cloning of fragment RS1' for construct CWP1KO1 (loxP site is underlined)
1872	CWP1KO1Pac rev 4	GC TTAATTAA TCC ATC AGC TCT ATT ATA TTA CCT GAA CG	cloning of fragment RS1' for construct CWP1KO1
1873	CWP1KO2 sbf for	GC CCTGCAGG GCCATTCCTGCCTCCACA	cloning of fragment RS2 for construct CWP1KO2
1874	CWP1KO2 xba rev	CG TCTAGA C CAT TGT GAC GTT GAA CCG AAA AG	cloning of fragment RS2 for construct CWP1KO2
1875	CWP1KO2 Xba for	GC TCTAGA ATAACTTCGTATAATGTGTACTATACGAAGTTAT GAAGCGCTGACCACAAATAAC	cloning of "floxed" *PAC* cassette for construct CWP1KO2 (lox5171 site is underlined)
1876	CWP1KO2 Nsi for	GC ATGCAT ATAACTTCGTATAATGTGTACTATACGAAGTTAT GTTCATGCCAAAAACGGGAAAAA	cloning of fragment RS2' for construct CWP1KO2 (lox5171 site is underlined)
1877	CWP1KO2 Pac rev	CG TTAATTAA AC CAA TAG TGC ATA TGT TTT GTT AAG A	cloning of fragment RS2' for construct CWP1KO2
1884	180BPupstrRS1for	CAGACTAGAATGCCCGAACA	amplification from 180bp upstream of region RS1
1885	180BPdnstrRS1'rev	ATA AAG ATG GTT CTA TAG TTA GCA G	amplification from 180bp downstream of region RS1'
1886	CWP1KO3SbfSwafor	GCC CTG CAG GAT TTA AAT GTC TTC CTC ATC ATA CGT CAT TAC C	cloning of fragment RS3 for construct CWP1KO3
1887	CWP1KO3Sph rev	CGG CAT GCG GTG TAG TGC CTT GTA TTC CAG TAT	cloning of fragment RS3 for construct CWP1KO3
1888	CWP1KO3Sph for	GCG CAT GCA TAA CTT CGT ATA AAG TAT CCT ATA CGA AGT TAT GAA GCG CTG ACC ACA AAT AAC	cloning of "floxed" *PAC* cassette for construct CWP1KO3 (lox2272$_d$ site is underlined)
1889	CWP1KO3Spe rev	CGA CTA GTG AAT TCG AGC TCG GTA CCA G	cloning of "floxed" *PAC* cassette for construct CWP1KO3
1890	CWP1KO3Spe for	GCA CTA GTA TAA CTT CGT ATA AAG TAT CCT ATA CGA AGT TAT GCA TGT AGA AGT CCA GAA AAT CCG	cloning of fragment RS3' for construct CWP1KO3 (lox2272$_d$ site is underlined)

1891	CWP1KO3SwaPacrev	CGT TAA TTA AAT TTA AAT TGG TGG ATG ACT TTC CAC CGT TTA	cloning of fragment RS3’ for construct CWP1KO3
1970	NEO1_seq_as	TCT TGA GTA GCC ACC AGT CC	amplification of *cwp1-4* from genomic DNA
2260	Nsi_NLS_Ala_NLS_Cas9_s	GC ATGCAT CCAAGAAAAAACGCAAGGTGGCTCCTAAGAAAAAGCGGAAAGTGGAC AAG AAG TAC AGC ATC GGC CTG G	cloning of *S. pyogenes* Cas9 with the insertion of a *Giardia* tandem nuclear localization signal
2261	Cas9_BglII_as	CG AGATCT CTTGCAGATAGCAGATCCGG	cloning of *S. pyogenes* Cas9
2262	BsaBI_Cas9_s	GC GATCAC CATCATGGAAAGAAGCAGCTTC	cloning of *S. pyogenes* Cas9
2263	Cas9_STOP_Pac_as	CG TTAATTAA TCA GGC ATA ATC GGG CAC GTC ATA GGG ATA	cloning of *S. pyogenes* Cas9
2310	cas9short_HA_stop_pac	CG TTAATTAA TCA CGCGTAGTCTGGGACATCGTATGGGTA GCTCAGGGGGGCCTTGGTGA	cloning of a truncated 1044bp variant of *S. pyogenes* Cas9
2407	Sbf_Pac_RS4N_for	GC CCTGCAGG TTAATTAA GCACGGGCTTGCTGACGA	cloning of fragment RS4N for construct CWP1 KO4
2408	Nsi_RS4N_rev	CG ATGCAT GTACCTGTAAGGCCCATGTC	cloning of fragment RS4N for construct CWP1 KO4
2412	Sbf_RS4N’_for	GC CCTGCAGG CCA TCC CAG AGG GTC TGT G	cloning of fragment RS4N’ for construct CWP1 KO4
2413	Sbf_Pac_RS4N’_rev	GC CCTGCAGG TTAATTAA AGG GGT CTT TTA GAA TTG ATG CGT	cloning of fragment RS4N’ for construct CWP1 KO4
2414	CWP1KO1chk_for	GC ATGCAT GCA AGA CAC TCG ACA AGA G	amplification of allele *cwp1-1* from genomic DNA
2415	CWP1KO1chk_rev	CG TTAATTAA TGC ATG AAG ATC AGT GCT AG	amplification of allele *cwp1-1* from genomic DNA
2416	CWP1KO2chk_for	GC ATGCAT ATGGACACAGAATGCACGAAG	amplification of allele *cwp1-2* from genomic DNA
2417	CWP1KO2chk_rev	CG TTAATTAA TCG TAC TCT CAG TAA CCT GTA C	amplification of allele *cwp1-2* from genomic DNA
2418	CWP1KO3chk_for	GC CCTGCAGG ATGGCCCAACAGCTTATAAG	amplification of allele *cwp1-3* from genomic DNA
2419	CWP1KO3chk_rev	CG TTAATTAA ATA ATC GTT TTC TCT GCG TTG	amplification of allele *cwp1-3* from genomic DNA
2426	Cre-HA-Pac-as	CG TTAATTAA CTA CGCGTAGTCTGGGACATCGTATGGGTA ATCGCCATCTTCCAGCAG	cloning of recombinant HA-tagged Cre recombinase (HA tag coding sequence is underlined)
2432	Hind_CWP1_s	GC AAGCTT CTAGCCACGCATGGGCTGT	cloning of the *CWP1* locus in pBluescript for complementation test
2433	CWP1_Spe_as	CG ACTAGT TCTTGAGTAGCCACCAGTCC	cloning of the *CWP1* locus in pBluescript for complementation test
2434	Not_CWP1p_LS_s	GC GCGGCCGC CTAGCCACGCATGGGCTGT	cloning of a recombinant *CWP2* locus in pBluescript for complementation test
2435	EcoRV_HA_CWP2_as	CG GATATC TCTTGAGTAGCCACCAGTCC	cloning of a recombinant *CWP2* locus in pBluescript for complementation test
2479	CWP2_961bp_fw	GGC AAT GCT AGC CGC TCT G	3' RACE: amplification step for *cwp2*
